# Experimental data supporting adaptive locomotor responses to salt stress in the mud-tidal gastropod populations (*Batillaria*)

**DOI:** 10.1016/j.dib.2021.107113

**Published:** 2021-04-30

**Authors:** Phuong-Thao Ho, Hoa Quynh Nguyen, Elizabeth M.A. Kern, Yong-Jin Won

**Affiliations:** a Institute of Fundamental and Applied Sciences, Duy Tan University, Ho Chi Minh City 700000, Vietnam; b Faculty of Natural Sciences, Duy Tan University, Danang City 550000, Vietnam; c Institute of Chemistry, Vietnam Academy of Science and Technology, Hanoi, Vietnam; d Division of EcoScience, Ewha Womans University, Seoul 03760, Republic of Korea; e Interdisciplinary Program of EcoCreative, Ewha Womans University, Seoul 03760 Republic of Korea

**Keywords:** adaptive divergence, invasive species, locomotion, salinity, snail behavior

## Abstract

This article describes the experimental locomotor data used to study the general and adaptive responses to salt stress of the northern Pacific intertidal gastropod *Batillaria attramentaria*. The data were obtained from a series of 30-day experiments on snails acclimated to different salinity regimes. Snails were collected from coastal areas on the eastern and western sides of the North Pacific Ocean. The data consist of three parts: 1) raw videos recording the locomotion of the snails when exposed to novel artificial salinity regimes in laboratory settings, 2) Spectral Time-Lapse results of movement distance of the snails extracted from the recorded videos, and 3) *CO1*-gene sequences isolated from individuals collected from four sampling sites. A Linear Mixed-effect Model inference procedure was applied in an attempt to assess the impacts of geographic distribution and genetic composition on the locomotor response to salt stress in the snail *B. attramentaria*. The locomotor dataset we present are the first reports of locomotor response to salt stress of the snail *B. attramentaria*, that is valuable for further exploration and understanding of the impacts of environmental changes on the physiology and adaptive capacity of living marine molluscs.

**Specifications Table**

**Table:** 

Subject	Biological sciences, Marine Biology, and Ecology
Specific subject area	Locomotor response and Adaptive locomotion
Type of data	Video Text Image
How data were acquired	Instruments: Sony NXCAM camera (AVCHD Progressive MPEG2 SD), AVS Video Editor v.7.1.2.262, Avidemux v.2.6.12, Spectral Time-Lapse (STL) toolbox, Matlab release R2014a
Data format	Raw (Videos, texts, and images deposited to Mendeley database) Secondary data: Obtained from raw data (Figures and Tables)
Parameters for data collection	The locomotor videos were produced by recording the snails’ movement when exposed to different artificial salinity regimes of 13, 23, 33, and 43 PSU. All snails were kept at 25 °C and a 12h Light:12h Dark photocycle.
Description of data collection	Snails were recorded for 1 hour every 2 days throughout 30-day acclimation experiments using Sony NXCAM camera (AVCHD Progressive MPEG2 SD). All videos were then saved in computer for further analyses. Later, a series of computer software such as AVS Video Editor v.7.1.2.262, Avidemux v.2.6.12, and Spectral Time-Lapse (STL) toolbox implemented in Matlab release R2014a were used to increase the video playback rate, crop videos, track the movement trail, and to measure the movement distances of the snails respectively. The final products of this process were locomotor videos, texts containing distance movement, and movement images which were deposited to the Mendeley database.
Data source location	City/Town/Region: Hacheon, Cheollabuk-do Country: South Korea Latitude and longitude for collected samples/data: 35 °32′N, 126 °33′Ex City/Town/Region: Nemuro, Hokkaido Prefecture Country: Japan Latitude and longitude for collected samples/data: 43 °15′N, 145 °28′E City/Town/Region: Matsushima Bay, Miyagi Prefecture Country: Japan Latitude and longitude for collected samples/data: 38 °22′N, 141 °4′E City/Town/Region: Monterey Bay, Elkhorn Slough, California Country: USA Latitude and longitude for collected samples/data: 36 °49′N, 121 °45′W
Data accessibility	With the article Repository name: NCBI Data identification number: MG241503-06 and MT800763 Direct URL to data: https://www.ncbi.nlm.nih.gov/popset?DbFrom=nuccore&Cmd=Link&LinkName=nuccore_popset&IdsFromResult=1527229734 Repository name: Dryad Data identification number: https://doi:10.5061/dryad.455mv2m Direct URL to data: https://datadryad.org/stash/dataset/doi:10.5061/dryad.455mv2m Repository name: Mendeley Data identification number: https://doi.org/10.17632/jjjmh26c2g.3 Direct URL to data: https://data.mendeley.com/drafts/jjjmh26c2g/3
Related research article	P.-T. Ho, H. Q. Nguyen, E. M. A. Kern, and Y.-J. Won, Locomotor responses to salt stress in native and invasive mud-tidal gastropod populations (Batillaria), Ecol. and Evol. 11, 458-470 (2020). https://doi.org/10.1002/ece3.7065

## Value of the Data

• These locomotor data present significant insights into the response to artificial salt stress of the intertidal gastropod *Batillaria attramentaria* in laboratory settings.
• These data could be utilized for further statistical analyses including a Linear Mixed-effect Model to study adaptive response to salt stress of introduced snails, which were accidentally introduced to a novel osmotic environment (Elkhorn Slough, CA, USA) approximately 80 years ago.
• These data are also valuable for further research on forecasting the impacts of environmental changes on the physiology and adaptive capacity of living marine molluscs.

## 1 Data Description

**Fig. 1:**
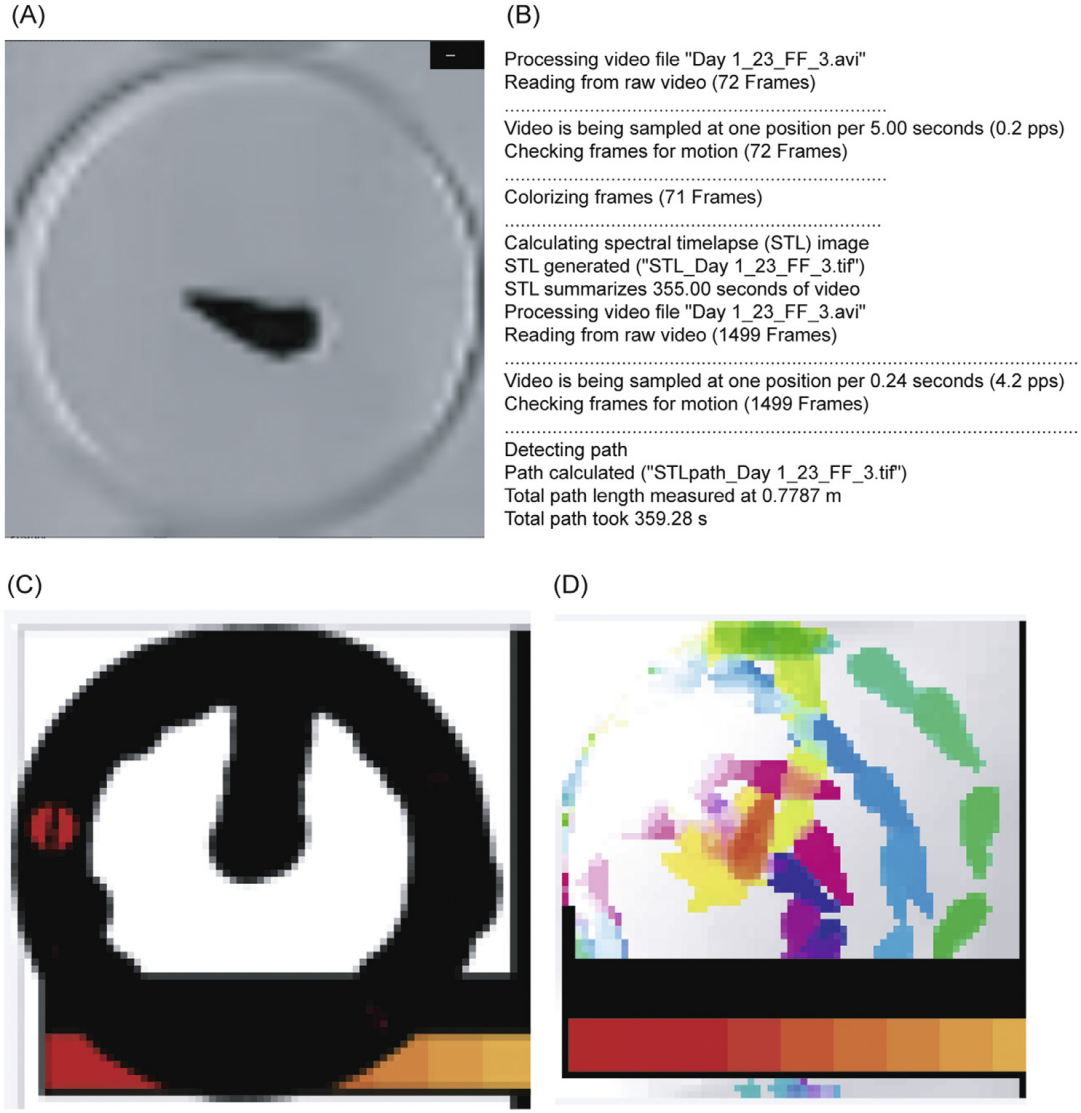
(A) An example screenshot of a locomotor video and Spectral Time-Lapse results of locomotor behavior tracking analyses of *Batillaria attramentaria*. (B) A text file (in .txt format) including locomotor video information, detecting path length and moving time. (C) A photo (in .tiff format) of moving trail of a snail. The red dot in figure C represents the initial position of the examined snail and the thick black circle is a moving trail of the snail recorded after the filming process. (D) Colored shapes of individual snails in figure D represent locations of an identical snail examined at different times. These different colors were according to the color map in the inset at the bottom of the figure. For the sake of simplicity and focusing on the individual snails, the colors of yellow, purple, navy, turquoise, and so on in the inset were cut off from it. The raw figure can be found in Mendeley repository database. (For interpretation of the references to color in this figure legend, the reader is referred to the web version of this article.)

The raw locomotor videos recorded snail movement during a 30-day period in which snails were exposed to different artificial salinity regimes. The intertidal snails *Batillaria attramentaria* (G. B. Sowerby I, 1855) were collected from coasts of the northeast and northwest Pacific Ocean. Sample collection details are provided in Table 1. The locomotor performance of the snail *B. attramentaria* in response to salt stress is presented as movement distance (.txt format file) and movement trails (.tiff format file) (Fig. 1); the former was to assess the impacts of geographic distribution and genetic composition on the locomotor responses of the snails.

**Table 1:** Sampling information of the *Batillaria attramentaria* examined in the present study.

Origin	Population	Location	Coordinates	*CO1* Genetic Lineage	Date of collection	Sea surface conditions
Native	Hacheon-ri	Cheollabuk-do, South Korea	35 °32′N, 126 °33′E	Tsushima	June, 2016	32±1 PSU, 24.5±0.5°C
Native	Nemuro city	Hokkaido Prefecture, Japan	43°15′N, 145 °28′E	Tsushima	June, 2017	31±2 PSU, 21.5±1.5 °C
Native	Matsushima Bay	Miyagi Prefecture, Japan	38 °22′N, 141°4′E	Kuroshio	May, 2018	31±2 PSU, 24±1 °C
Introduced	Monterey Bay	Elkhorn Slough, California, The USA	36 °49′N, 121 °45′W	Kuroshio	February, 2017	4 PSU, 6±1 °C

**Fig. 2:**
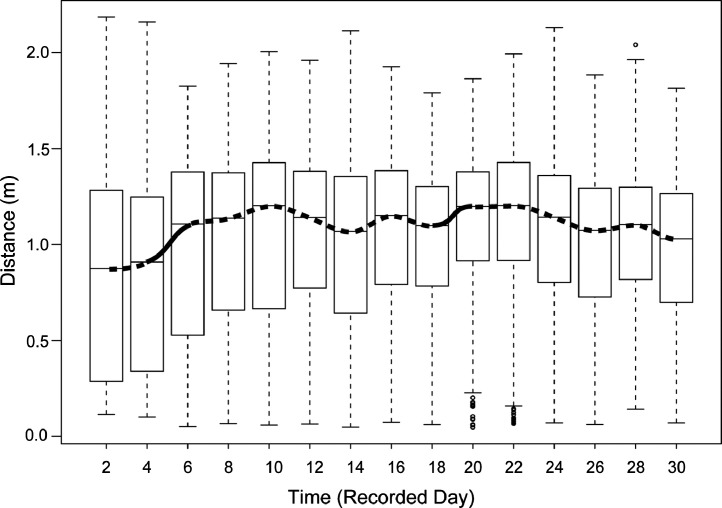
Generic time-response function of the movement distance of *B. attramentaria*. The predicted variable-response derived from the linear mixed-model test, specifying random intercept for subject, with *N* = 280 individuals split into groups of snails observed over 30 days. The solid and dashed black curves represent the statistically significant and insignificant difference between two means, respectively. The bottom and top of the box are the 25^th^ and 75^th^ percentile of the movement distance, the straight dash lines show the 50^th^ percentile, and the ends of the whiskers represent the minimum and maximum estimates of the movement distance. Outliers are represented by black circles beyond the whiskers.

**Fig. 3:**
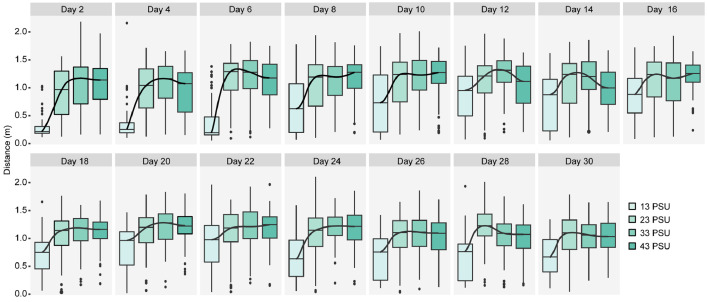
Generic Salinity Expose-response function of the movement distance of *Batillaria attramentaria* under the effect of time. Gradient, turquoise-colored boxes represent the movement distance of snails exposed to different salinities. The solid black curves connect all means of movement distance of the snails exposed to different salinities. The bottom and top of the boxes are the 25^th^ and 75^th^ percentiles, the dashed vertical lines show the 50^th^ percentiles, and the ends of the whiskers represent the minimum and maximum estimates of the movement distance. Outliers are represented by black circles beyond the whiskers.

The data comprise three geographic variables (origin o, location lo, and population p), one genetic variable (*CO1* genetic lineage li), and one temporal variable time t (video recording date). Ho et al. [1] found that *B. attramentaria* acclimated to an extremely low salinity of 13 PSU exhibited significantly shorter movement distance than individuals acclimated to normal or moderately altered salinities of 23, 33, and 43 PSU [Supplemental Table A2 and Fig. 3A in [2]. In addition, the introduced snails, which were accidentally introduced to north America [Monterey Bay, Elkhorn Slough, CA, USA, [3,4], exhibited shorter movement distance than those from native habitats [Supplemental Table A2 and Fig. 3B, C, D in [2]. These findings suggest that the introduced snails may have adapted or acclimated to the novel osmotic conditions of their new habitat, where salinity fluctuations are wider than their native habitats. Here, we present how snails responded over the courses of 30-day experiments by applying a Linear Mixed-effect Model (LMM) to movement distance. We discovered that snails significantly changed their locomotor performance after 30 days with *F*_Time_ (1,14) = 12.42 and *P*-value < 0.0001 (Table 2 A and Fig. 2). Means of movement distance of the snails recorded every two days over a 30-day period are presented in Table 2 B and Fig. 2. Subsequent Tukey post-hoc tests revealed that snails significantly increased their movement distance over the experiment period with d_Day 30-Day 2_ = 0.12 m ± 0.031 and *P*-value < 0.005 (Table 3). On the other hand, we also observed that significant increases in locomotion occurred between two pairs of consecutive recording dates, Day 4 vs. Day 6 and Day 18 vs. Day 20 (Table 3 and Fig. 2). This result is graphically presented in Fig. 3, which shows the trend of movement distance change between groups of snails experimentally exposed to different salinities.

**Table 2:** (A) Summary of the analysis of variance to assess the effects of the explanatory variable of time on the movement distance of the snail *Batillaria attramentaria*. (B) Mean movement distance of *B. attramentaria* recorded every 2 days during the 30-day experiment.

(A)					
		N.d.f	D.d.f	*F*-value	P-value
Intercept		1	3827	2952.95	<0.0001
Time		14	3827	12.42	<0.0001

N.d.f: Numerator degree of freedom, D.d.f: Denominator degree of freedom.					
(B)					

Time	Sample size (*N*)	Estimate (meter)	Standard Error	Lower CL	Upper CL

Day 2	280	0.83	0.027	0.78	0.88
Day 4	280	0.84	0.027	0.79	0.89
Day 6	280	0.96	0.027	0.90	1.01
Day 8	280	1.01	0.027	0.95	1.06
Day 10	280	1.05	0.027	1.00	1.10
Day 12	280	1.06	0.027	1.00	1.11
Day 14	280	0.99	0.027	0.93	1.04
Day 16	280	1.06	0.027	1.01	1.12
Day 18	280	1.02	0.027	0.97	1.06
Day 20	280	1.12	0.027	1.06	1.17
Day 22	280	1.12	0.027	1.06	1.17
Day 24	280	1.04	0.027	1.00	1.10
Day 26	280	0.97	0.027	0.92	1.02
Day 26	280	1.01	0.028	0.96	1.07
Day 30	280	0.95	0.028	0.90	1.01

CL: Confidence Level

**Table 3:** Tukey post-hoc test of the Linear Mixed-effect models for predictor of the time variable. This data are visually illustrated in Fig. 2.

Contrast	Estimate (meter)	Standard Error	D.f	*t*-ratio	*P*-ratio
Day 2–Day 4	−0.01	0.26	3827	−0.39	1.0000
Day 4–Day 6	−0.12	0.03	3827	−4.61	0.0004⁎⁎
Day 6–Day 8	−0.05	0.03	3827	−1.88	0.8641
Day 8–Day 10	−0.04	0.03	3827	−1.70	0.9333
Day 10–Day 12	−0.01	0.03	3827	−0.30	1.0000
Day 12–Day 14	0.07	0.03	3827	2.79	0.2526
Day 14–Day 16	−0.08	0.03	3827	−3.02	0.1452
Day 16–Day 18	0.04	0.03	3827	1.63	0.9529
Day 18–Day 20	−0.09	0.03	3827	−3.66	0.0205*
Day 20–Day 22	0.00	0.03	3827	0.01	1.0000
Day 22–Day 24	0.07	0.03	3827	2.83	0.2414
Day 24–Day 26	0.07	0.03	3827	2.83	0.2316
Day 26–Day 28	−0.04	0.03	3827	−1.56	0.9671
Day 28–Day 30	0.06	0.03	3827	2.12	0.7198
Day 2–Day 30	−0.12	0.03	3827	−4.08	0.0042⁎⁎

^⁎⁎⁎^ indicates *P* < 0.001.

⁎⁎ indicates 0.001 < *P* < 0.01.

⁎ indicates 0.01 < *P* < 0.05.

## 2 Experimental Design, Materials and Methods

### 2.1 Sample collection

Samples of the intertidal *Batillaria attramentaria* snails were collected from Hacheon-ri, Cheollabuk-do, South Korea (100 individuals); Nemuro city, Hokkaido Prefecture, Japan (100 individuals); Matsushima Bay, Miyagi Prefecture, Japan (100 individuals); and Monterey Bay, Elkhorn Slough, California, USA (50 individuals) (Table 1). All samples were kept alive and transferred to the Molecular Evolution laboratory, Division of EcoScience, Ewha Womans University, Seoul, South Korea for the salinity experiments.

### 2.2 Salinity experiments

Snails were kept at 25 °C and on a 12h Light:12h Dark photocycle for two days to reduce any effects of transportation. After this acclimation period, we conducted 30-day experiments on the four collected sets of samples separately. Snails were randomly equally divided into five groups with 20 individuals per group for the native-population samples and 10 individuals per group for the introduced-population sample. Five groups of each population were cultured separately in plastic aquaria [40 × 23 × 21 cm^3^, with an inclined layer of sea sand on the bottom and fresh aerated artificial sea, Supplementary Figure S1 in [5] at different salinities of 13, 23, 33, and 43 PSU for 30 days. We marked each of the snails in each group with different colors of nail polish (Innisfree, ROK). All individuals were fed with excised fresh seaweed (Ottogi, ROK) every two days throughout the experiments.

### 2.3 Locomotion recording and analyzing

We recorded snails and tracked their movement for one hour every two days from 9:00 to 15:00 throughout the laboratory experiments. To monitor the snails’ performance and track their movement distance, we placed each snail in the center of a single disposable Petri dish which was filled with artificial seawater and recorded them using a Sony Nxcam (AVCHD Progressive MPEG2 SD). Snails from the same group were recorded at the same time. The camera was mounted on a tripod, and the camera was situated above the twenty Petri dishes to record all the dishes at once. Saline water was freshly prepared with overnight-aerated distilled water and Instant Ocean Sea Salt (United Pet Group Inc., Cincinnati, OH, USA). After being recorded, all snails were conveyed back to their corresponding aquaria. All recorded videos were uploaded to a computer for computational analyses. Video analyses were then conducted following the protocol described in our earlier study [5]. Briefly, we used a series of software packages such as AVS Video Editor v.7.1.2.262 to increase the play-back rate of the recorded videos, Avidemux v.2.6.12 to crop videos, and Spectral Time-Lapse (STL) toolbox [6] implemented in Matlab release R2014a (MathWorks Inc., Natick, Massachusetts, USA) to estimate the movement distance of snails. Movement distance of the snails was measured in meter.

### 2.4 Statistical analyses

A Linear Mixed-effect Model (LMM) was employed to (1) assess the impacts of salinity exposure (es), geographic distribution (g: population p, location lo, and origin o), and *CO1* genetic lineage (li), as well as their interactions (es × o, es × o + li, es × lo, es × lo + li, es × p, es × p + li, es × li, es × li + o, es × li + lo, and es × li + p) on the snails’ locomotion, and (2) model the snails’ movement distance over time due to salinity stress. In the analysis, es refers to the salinity level used in the laboratory experiments including four levels: 13, 23, 33, and 43 PSU; g indicates the geographic location of each population p (Hacheon in Korea, Nemuro city and Matsushima bay in Japan, and Elkhorn Slough in the USA); location lo (Korea, Japan, or the USA); origin o (native or introduced); and *CO1* lineage li is defined as either Tsushima (comprising the Hacheon and Nemuro populations) or Kuroshio (comprising the Matsushima and Elkhorn Slough populations) based on the individual's position in a *CO1* phylogenetic tree. Subsequently, we conducted multimodel inference and model averaging [7] to select the best-fit model that best described snail locomotor response using the corrected Hurvich and Tsai's Criterion (AICC). The models were compared based on AICc values using the MuMIn package MuMIn 1.9.13 [8] for R 3.0.2. The model with the lowest AICc value and those satisfying a ΔAICc ≤ 6 cut-off rule [9] were considered the best-fit models. Following the multimodel inference procedure, we conducted post-hoc multiple comparison tests of the models to examine the effects of each explanatory factor. Additionally, we used MuMIn to perform model averaging and estimate the importance of predictor variables by summing the weights of models where the variables appeared. The significance level was set at α = 0.05 for all statistical tests.

For the present study, we additionally conducted LMM to the movement distance data in order to assess the impact of temporal variable time t on the response to salt stress of the snails. Subsequently, we conducted Tukey post-hoc tests to examine the difference in movement distance of the groups of snails between dates over the course of acclimation experiments.

### 2.5 Mitochondrial CO1 gene sequencing

After acclimation experiments, we extracted genomic DNA using a Dneasy Blood & Tissue kit (Qiagen, Hilden, Germany) from fresh foot tissue of all experimental snails from Korea, Japan, and the USA, followed by PCR (Fastmix/Frenchetm PCR kit, Cat. No. 25401, IntronBio, ROK) using published mitochondrial COI primers [10]. PCR products were purified using a Dr. Prep kit (Cat. No. MD108P, MGmed, ROK). Sequencing reactions were performed using a Bigdye Terminator V3.1 Cycle Sequencing kit (Bionics, ROK).

## Ethics Statement

We confirm that all experiments comply with the ARRIVE guidelines and were be carried out in accordance with the U.K. Animals (Scientific Procedures) Act, 1986 and associated guidelines, EU Directive 2010/63/EU for animal experiments, or the National Institutes of Health guide for the care and use of Laboratory animals (NIH Publications No. 8023, revised 1978).

## CRediT Author Statement

**Phuong-Thao Ho:** Conceptualization, Data curation, Formal analysis, Methodology, Writing – original draft, Writing – review and editing; **Hoa Quynh Nguyen:** Formal analysis; Writing–review and editing; **Elizabeth MA Kern:** Writing – original draft, Writing – review and editing; **Yong-Jin Won:** Funding acquisition, Writing – review and editing.

## Declaration of Competing Interest

The authors declare that they have no known competing financial interests or personal relationships which have or could be perceived to have influenced the work reported in this article.
